# Supplementary material for: Mending cracks atom-by-atom in rutile TiO2 with electron beam radiolysis
Source: Nat Commun. 2023 Sep 26;14:6005. doi: 10.1038/s41467-023-41781-x (PMC10522652; doi:10.1038/s41467-023-41781-x)
Supplement: Supplementary file 1 — Supplementary Information [file 41467_2023_41781_MOESM1_ESM.pdf]

**Supporting Information**

# Mending Cracks Atom-by-atom in Rutile $TiO_2$ with Electron Beam Radiolysis

Silu Guo[1], Hwanhui Yun[1, 2], Sreejith Nair[1], Bharat Jalan[1] and K. Andre Mkhoyan[1]*

[1]*Chemical Engineering and Materials Science, University of Minnesota, Twin Cities, Minneapolis, MN 55455, USA*

[2]*Korea Research Institute of Chemical Technology, Daejeon 34114, Korea*

*Corresponding author

Email: mkhoyan@umn.edu

This PDF file includes:

Supplementary Figures 1 to 15

Supplementary Tables 1 and 2

Captions to Supplementary Movies 1 to 3

Supplementary references

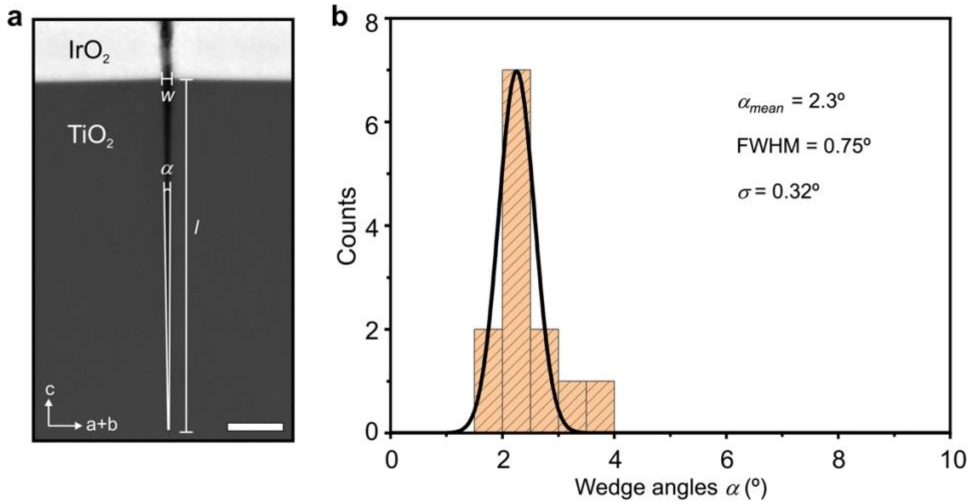


**Supplementary Figure 1. Statistics of the crack wedge angles. a**, HAADF-STEM image of a typical crack in rutile $TiO_2$ formed due to epitaxial stresses introduced by a rutile-$IrO_2$ film grown on it by SSMO-MBE. The crack can be described either by length *l* and width *w* (measured at the $TiO_2$/$IrO_2$ interface), or by wedge angle $\alpha$. scale bar = 20 nm. **b,** Histogram of wedge angles based on measurements from 12 cracks. The histogram is fitted with a Gaussian function, which shows a narrow distribution with a peak position at 2.3º. The full width half maximum (FWHM) of the Gaussian function is 0.75º and the standard deviation $\sigma$ is 0.32º.

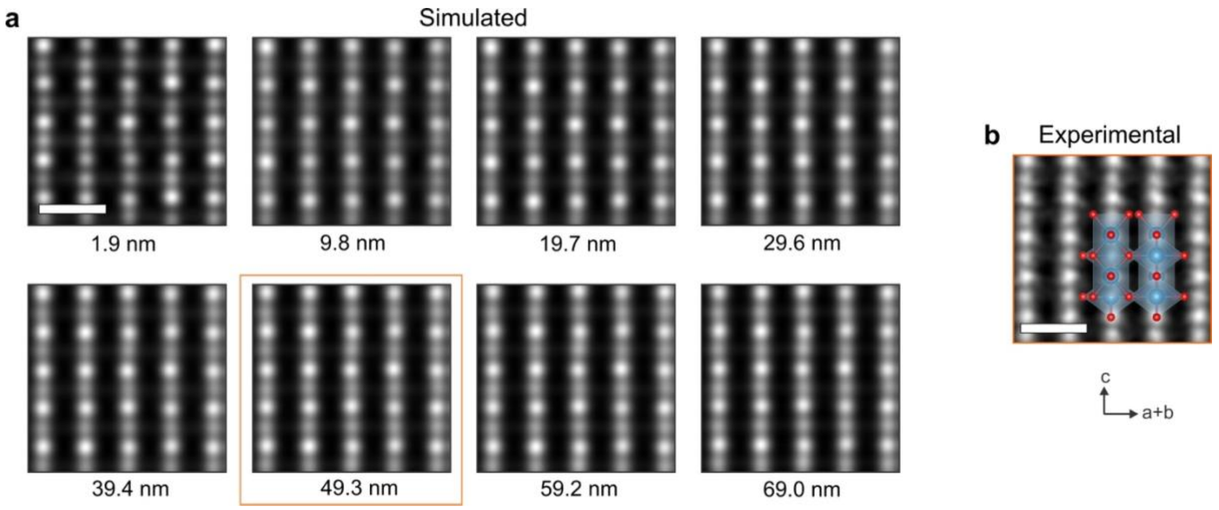


**Supplementary Figure 2. Simulated HAADF-STEM images of rutile-$TiO_2$ crystal viewed in the [110] direction. a,** Simulated atomic-resolution HAADF-STEM images of rutile-$TiO_2$. Sample thicknesses ranging from 1.9 nm to 69.0 nm are considered. These images are simulated using *Multislice* method and TEMSIM code developed by Kirkland[1] with following STEM parameters: $E_0$ = 200 keV, $C_{S(3)} = 0$, $\triangle f = 0$, $\alpha_{obj}$ = 25 mrad, and HAADF detector inner and outer angles of 50 and 200 mrad. These values are used to mimic the condition of the STEM used for these experiments. Thermal atomic displacement values of 0.075 Å for Ti and 0.110 Å for O atoms are used.[2,3] Ten frozen phonon configurations are averaged for each image. The final simulated images were convoluted with 2D Gaussian function with the full width at half maximum (FWHM) of 1 Å to incorporate the effects of source size.[4] Scale bar is 0.5 nm. **b,** Experimental atomic-resolution HAADF-STEM image of rutile-$TiO_2$ crystal in the same [110] orientation obtained from region next to a crack. Image is low-pass filtered for noise reduction. Atomic model of rutile-$TiO_2$ is overlayed on the image to show the relationships between columns of Ti atoms and columns of Ti and O atoms and their HAADF "dim" and "bright" intensities, correspondingly. Relative intensity comparison suggests that the experimental image is from about $t$ = 50 nm thick region. Scale bar is 0.5 nm.

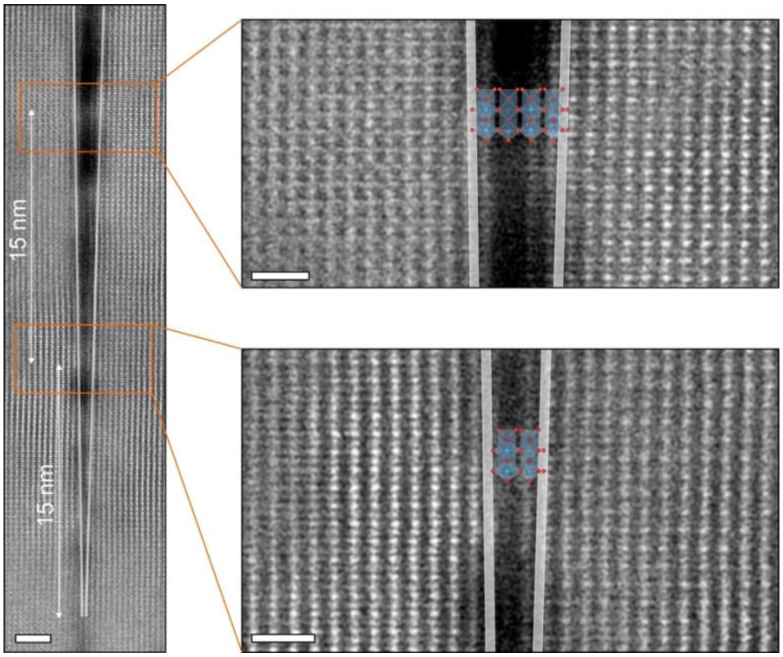


**Supplementary Figure 3. Closer look at the bridging sites of the crack after initial electron beam exposure.** HAADF-STEM image taken after 1.5×10$^{6}$ e Å$^{-2}$ electron doses. This is the same image shown in Figure 3 of the main text. Scale bar is 2 nm. Two magnified atomic-resolution images of the bridging regions of the crack are also shown. Formation of rutile $TiO_2$ at these locations, where the gaps are an integer number of crystal unit cells (top region with $2a_{[110]}$ and bottom region with just $a_{[110]}$), can be seen. Scale bars are 1 nm. Atomic models of rutile $TiO_2$ are overlayed on these images for guidance. Images are low-pass filtered for noise reduction.

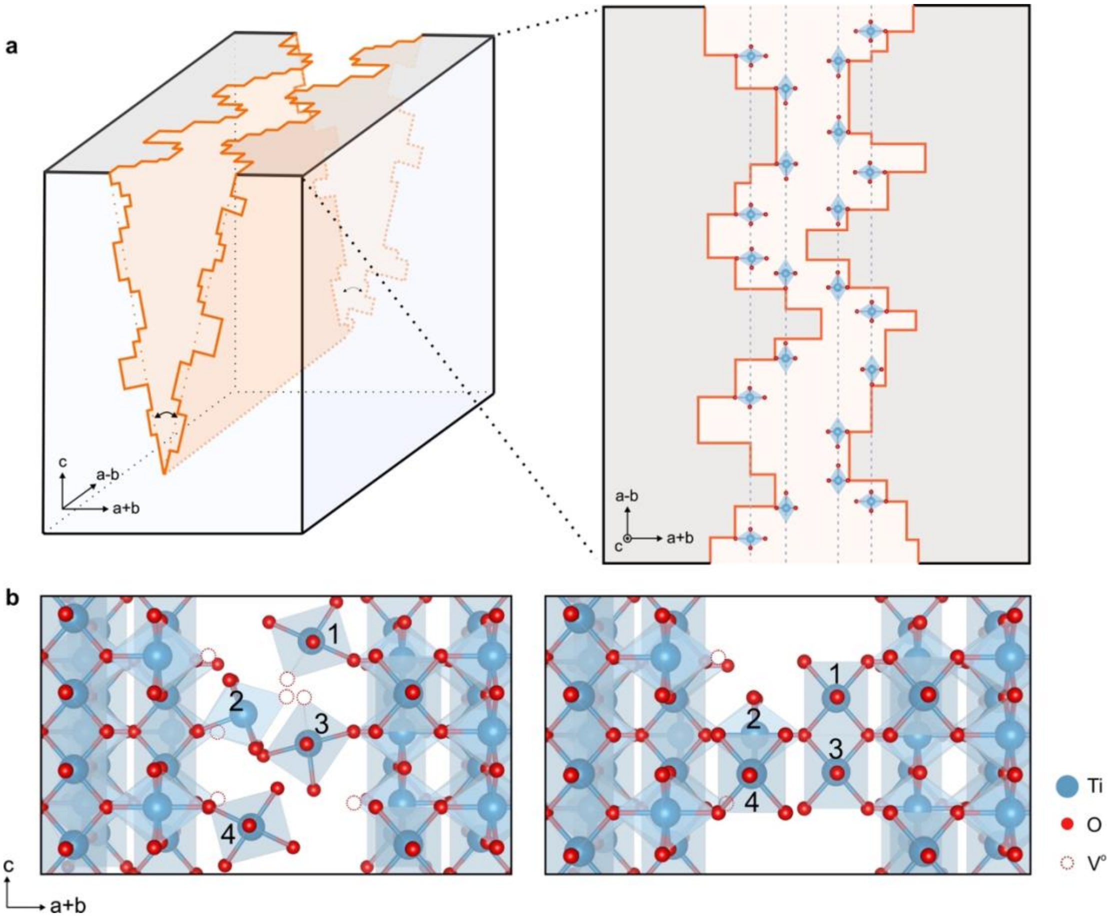


**Supplementary Figure 4. Simplified 3D models of the cracks at different stages. a,** (left) 3D model and (right) top view of the crack at early stage of mending illustrating initial structural changes with "octahedra" units at the edges and corners. **b,** Simplified 3D schematics showing locking of "octahedra" units from opposite sides of the crack. The dashed open red circles are missing oxygens. After locking "octahedra" 1 shares its left-bottom oxygen with "octahedra" 2 and 3, resulting in all units having 6 oxygens and turning them into true $TiO_6$ octahedrons.

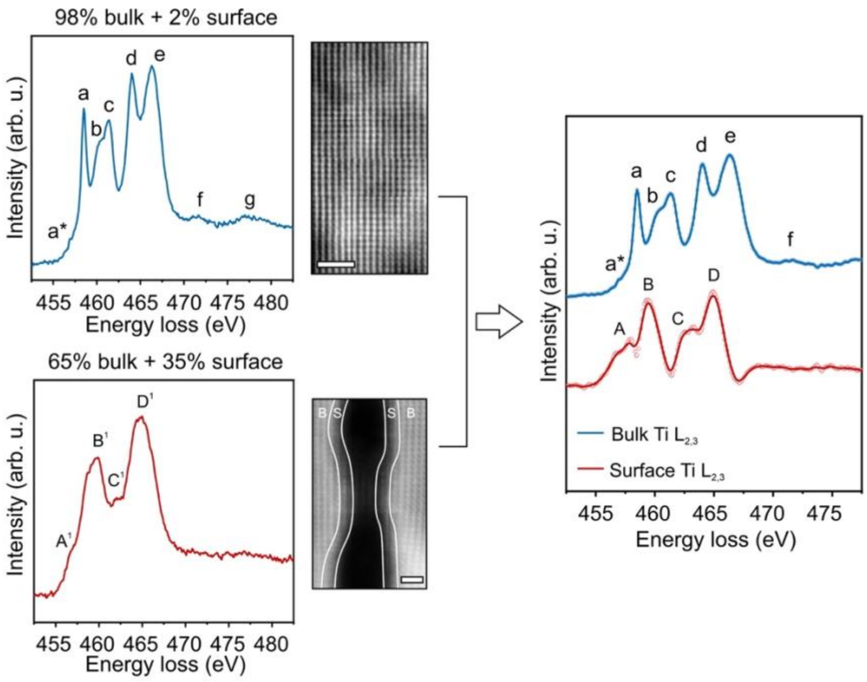


**Supplementary Figure 5. Determination of reference bulk and surface EELS Ti $L_{2,3}$-edges.** Monochromated EELS Ti $L_{2,3}$-edges are obtained from two locations of the sample. One from the region in-between the cracks ($I_1$) and one from the edge region of the crack ($I_2$). The spectrum $I_1$, shown in top left, based on an evaluated thickness of the sample in this region $t$ = 52 nm, should consist of 98% bulk and 2% surface Ti $L_{2,3}$-edges. The spectrum $I_2$, shown in bottom left, based on evaluated thickness of the sample in this region $t$ = 52 nm and wedge surface fraction relative to bulk as indicated in the HAADF-STEM image on the right ("S" for surface and "B" for bulk), should consist of 65% bulk and 35% surface Ti $L_{2,3}$-edges. Scale bars are 2 nm and 1 nm, correspondingly. Using these two spectra as a "a system of two equations with two unknowns", the reference spectra of bulk and surface EELS Ti $L_{2,3}$-edges were determined. They are shown on the right.

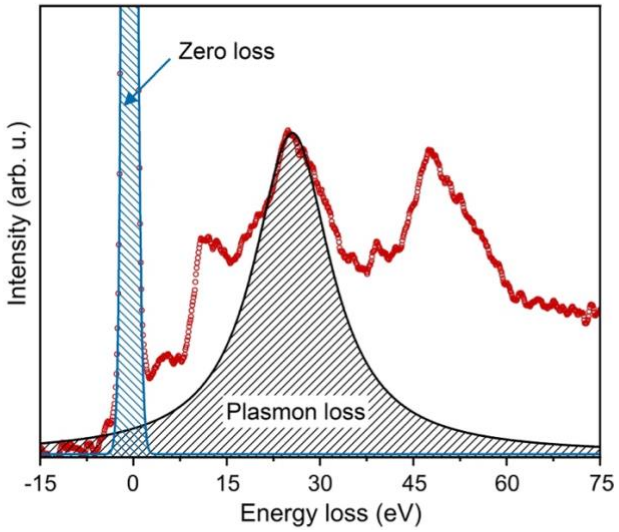


**Supplementary Figure 6. Thickness determination using low-loss EELS.** For thickness determination of the specific location of the rutile-$TiO_2$ sample, first, low-loss EELS spectrum was obtained from that region. Then, the "log-ratio" method was applied.[5] The mean free path of plasmon excitation in rutile $TiO_2$ is $\lambda_p$ = 130 nm.[6] The zero-loss peak is fitted by a Gaussian function and the plasmon peak is fitted by a Lorentzian function after zero-loss peak was subtracted.[7] This method was used to evaluate the thickness of the regions discussed in Supplementary Figure 4.

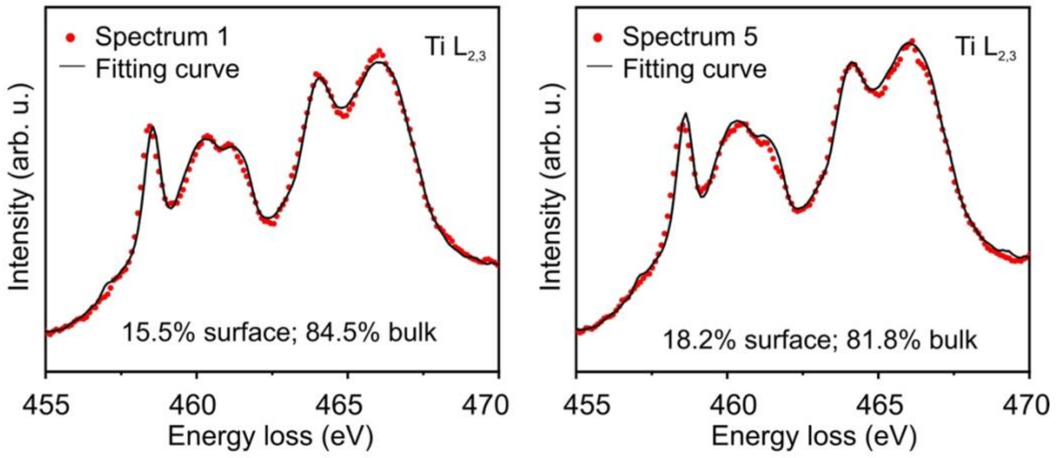


**Supplementary Figure 7. Determination of relative fractions of bulk and surface components in measured EELS Ti $L_{2,3}$-edges.** The fractions of surface and bulk components in each EELS Ti $L_{2,3}$-edge spectrum, discussed in Figure 4a in the main text, are determined by fitting it with a linear superposition of two reference spectra: $I = x \cdot I_s + (1 - x) \cdot I_b$. Here $I_b$ and $I_s$ are the reference spectra of surface and bulk Ti $L_{2,3}$-edges, respectively, determined earlier (Supplementary Figure 4). Through "least square" fitting algorithm, the normalized fractions of surface and bulk components are identified for each spectrum. Two examples of such analysis performed on Spectrum 1 and Spectrum 5 from Figure 4a in the main text are shown here.

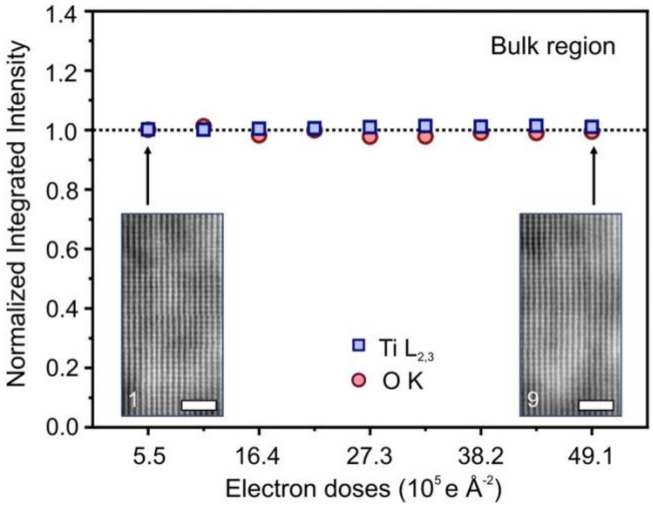


**Supplementary Figure 8. EELS analysis of bulk region in rutile $TiO_2$.** HAADF-STEM images with parallel EELS measurements from bulk region of the rutile $TiO_2$ showing no identifiable compositional and structural changes. Scale bars are 2 nm. The changes in the number of Ti and O atoms as a function of electron dose in beam-exposed area are evaluated using integrated intensities of Ti $L_{2,3}$- and O K-edge EELS spectra similar to those discussed in Figure 3d in the main text. The irradiation doses used here are comparable with those used for crack regions (see Figures 3c and 3d in the main text).

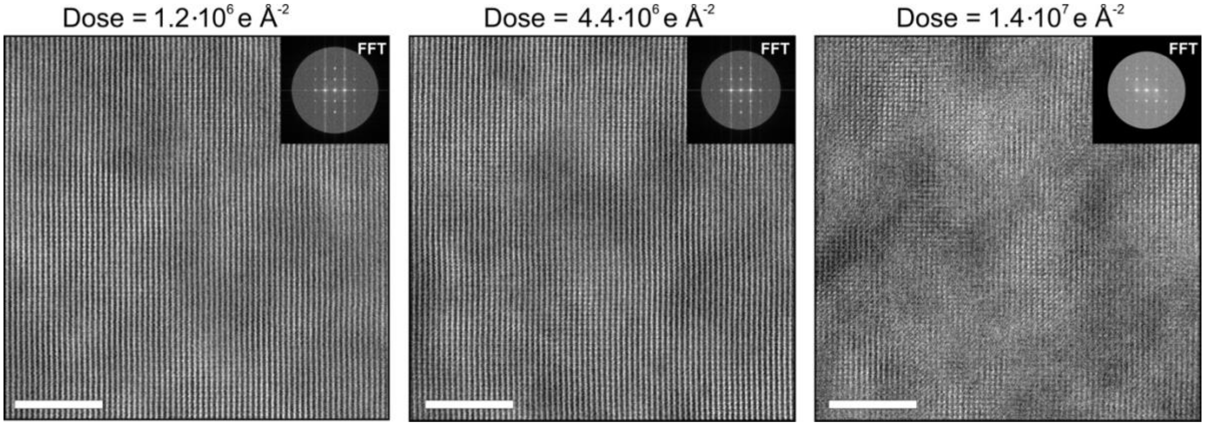


**Supplementary Figure 9. Effects of the high electron beam doses in bulk region of rutile $TiO_2$.** Atomic-resolution HAADF-STEM images of bulk region of the rutile $TiO_2$ at three e-beam exposure doses. The FFTs are shown in the upper-right corner. Partial loss of crystallinity is visible in the image at electron doses $1.4\times10^7$ e $Å^{-2}$. Images are low-pass filtered for noise reduction. Scale bars are 5 nm.

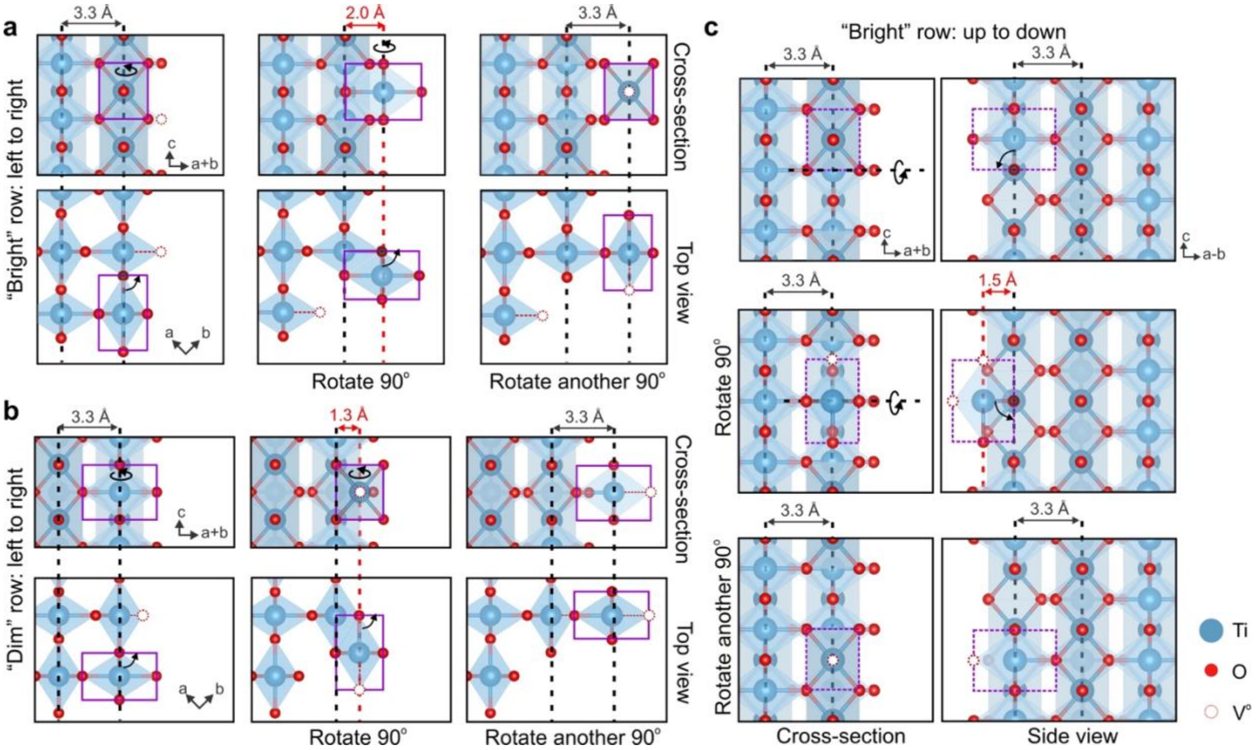


**Supplementary Figure 10. A rotational model for $TiO_6$ octahedral units. a,** Illustration of the "2-step rolling" model showing how an octahedral unit (highlighted in purple) at the edge of the crack from the "bright" atomic columns can rotate 90º and occupy an interstitial site in first step and then reach its final stable site after another 90º rotation in second step. The axes of rotations are indicated. Both cross-sectional ([110] projection) and top ([001] projection) views are shown. **b,** The "2-step rolling" model now illustrated for octahedral unit from the "dim" atomic columns. Unique interstitial sites occupied by rolling $TiO_6$ "octahedra" after the first 90º rotation can be characterized by their characteristic distances from the neighboring atomic column (highlighted by the red dashed line). **c,** "2-step rolling" model showing how an octahedral unit (highlighted in purple) at the edge of the crack from the "bright" atomic columns can rotate 90º down (or up) along the crack line and occupy an interstitial site in first step and then reach its final stable site after another 90º rotation in second step. The axes of rotations are indicated. Both cross-sectional ([110] projection) and side ([1$\bar{1}$0] projection) views are shown. Unique interstitial site occupied by rolling $TiO_6$ octahedra after first 90º rotation can be characterized by its distance from the neighboring atomic column (highlighted by the red dashed line). The red dashed open circles in models are oxygen vacancies, $V^O$.

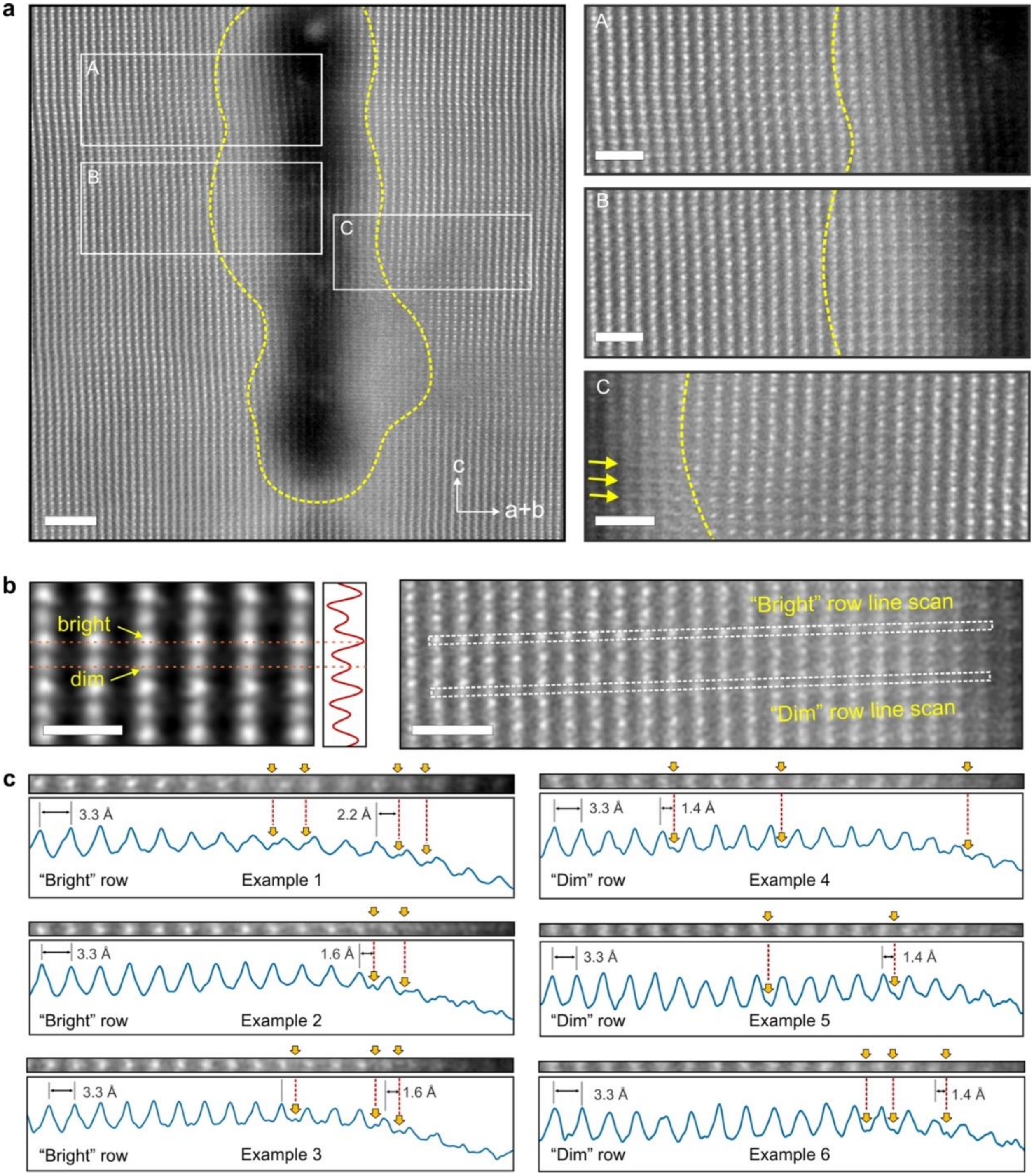


**Supplementary Figure 11. HAADF-STEM imaging of a recrystallized crack for interstitial site analysis. a,** Atomic-resolution HAADF-STEM image of a wider crack region after $4.1\times10^{6}$ e $Å^{-2}$ electron beam exposure. The yellow dashed line indicates the boundary between considerably modified regions next to crack and the rest of the rutile-$TiO_2$ sample. The modified regions clearly show different lattice contrast compared to original rutile $TiO_2$. Scale bar is 2 nm. Magnified images from these regions, including bridging and non-bridging sites (labeled A to C), show close-up of these structural modifications. For example, C shows how "bright" rows become less bright

with added intensities between original rutile columns, indicated by yellow arrows. Scale bars are 1 nm in A, B and C. **b,** Atomic-resolution HAADF-STEM image of rutile $TiO_2$ viewed along [110] direction. Orange dashed lines show the rows of “bright” and “dim” atomic columns. The “bright” spots corresponding to atomic columns with Ti and O atoms and “dim” spots to columns with only Ti atoms. Scale bar is 0.5 nm. On the right, a magnified image from the crack bridging region with dashed rectangle strips indicating where the “Bright” row and “Dim” row line scans are taken. Scale bar is 1 nm. **c,** Six examples of one-atom-width strips of HAADF-STEM images from the larger image shown in **a**, and the corresponding intensity line scans. They show the locations of Ti atoms at interstitial sites with specific 2.2 Å, 1.6 Å and 1.4 Å spacings from the main columns. Here “bright” rows are in examples 1 to 3 and “dim” rows in examples 4 to 6. The statistical analysis of these interstitial locations is presented in Figure 4d in the main text. Images are low-pass filtered for noise reduction.

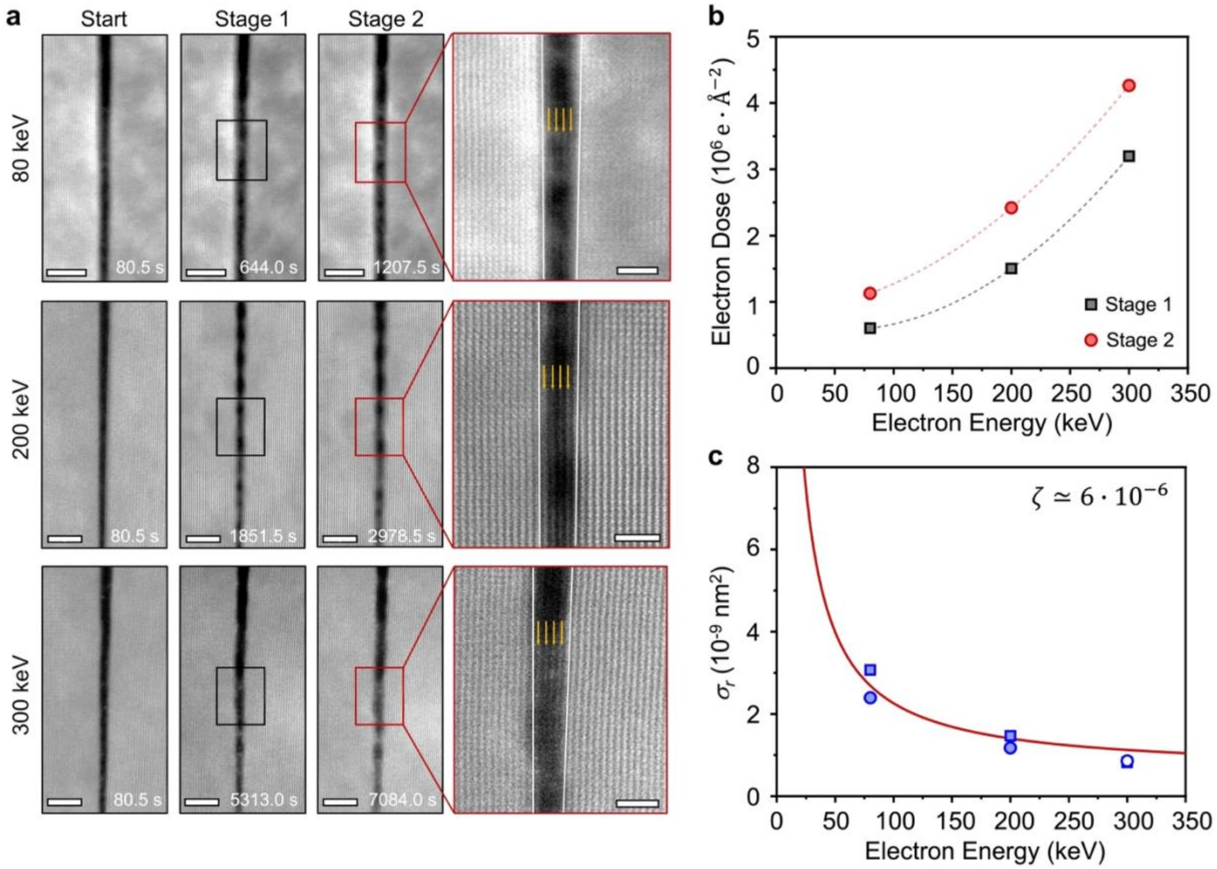


**Supplementary Figure 12. Radiolysis under three electron beam energies: 80 keV, 200 keV and 300 keV. a,** HAADF-STEM images of three cracks in the same rutile $TiO_2$ sample. They were exposed to STEM electron probes with beam energies of $E_0$ = 80, 200 and 300 keV, correspondingly. Scale bars are 5 nm. Magnified (in red box) regions show 2 unit-cells-wide rutile $TiO_2$ is formed in the crack. Scale bars are 2 nm in the magnified regions. The images are low-pass filtered. The exposure times needed for two gap restructuring stages are indicated on the images, respectively. The thickness of the filled region in the gap is estimated to be about 10 nm (stage 1) and 15 nm (stage 2). **b,** Electron doses needed to form the rutile $TiO_2$ of same thicknesses in the gap (based on HAADF intensity) for both stages are plotted for all three electron beam energies. **c,** The data from panel **b** is converted into probabilities of single radiolysis-driven event (one-step of rolling) and fitted to a function describing the cross-section of radiolysis:[8] $\sigma_r(E_0) = 8\pi a_0^2 \times \left(Z\frac{U_R}{m_0c^2}\right)\left(\frac{U_R}{E_{th}\beta^2}\right) \times \zeta$, where $a_0$ is the Bohr radius, $Z$ is the number of moving unit, $U_R$ is Rydberg energy, $m_0$ is the rest mass of electron, $c$ is the speed of light, $E_{th}$ is the threshold energy that must be transferred to produce a movement, and $\beta = \frac{\mathrm{v}}{\mathrm{c}} = \sqrt{1 - \left(1 + \frac{E_0}{m_0c^2}\right)^{-2}}$. Considering that, for rolling an octahedral unit, it will require breaking about 1-3 bonds (see Tables S1 and S2), the efficiency factor is estimated to be $\zeta \simeq 6 \times 10^{-6}$. For more accuracy, the fitting was performed only on results from $E_0$ = 80 and 200 keV probes.

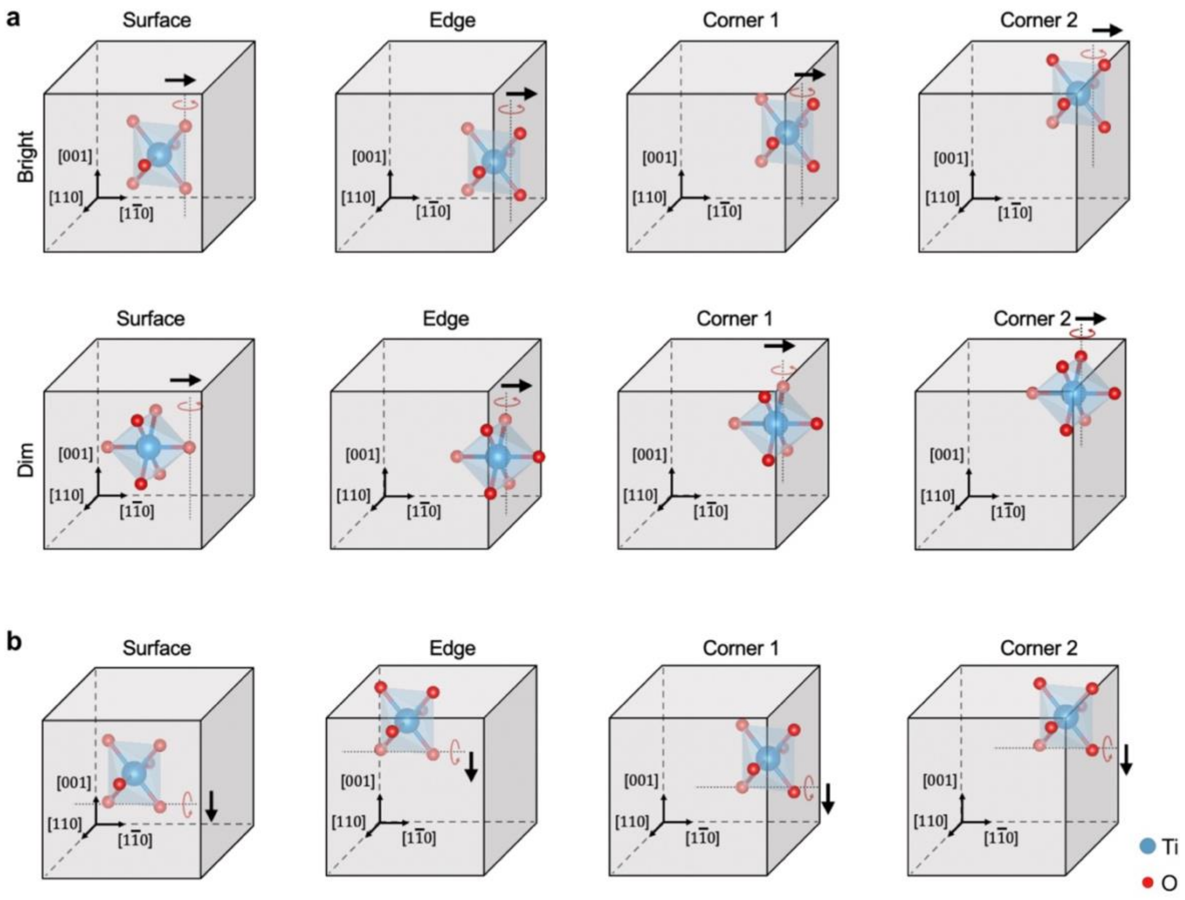


c

| | Surface | | Edge | | Corner 1 | | Corner 2 | |
|---|---|---|---|---|---|---|---|---|
| Rolling Direction | Left-Right | Up-Down | Left-Right | Up-Down | Left-Right | Up-Down | Left-Right | Up-Down |
| Dangling bonds | 2 (b)<br>2 (d) | 2 | 4(b)<br>4(d) | 7 | 5 (b)<br>6 (d) | 5 | 8 (b)<br>8 (d) | 8 |
| Bond-breakage | 3-6 (b)<br>5-8 (d) | 3-6 | 4-6 (b)<br>3-4 (d) | 1 | 2-3 (b)<br>2-3 (d) | 1 | 2-3 (b)<br>2-3 (d) | 1 |

**Supplementary Figure 13. Models of possible surface, edge and corner octahedrons rolling.** **a,** Models of two types of octahedra rolling from left to right (or from right to left) along [110] direction. The first row corresponds to "bright" row and the second to "dim" row (described in Supplementary Figure 9a). The orientation of these models is the same as the sample with crack. **b,** Models of octahedral unit rolling from up to down (or from down to up). Black arrows indicate octahedron rolling directions. **c,** A summary table of the numbers for the dangling bonds and bond-breakages needed for rolling of the octahedral units shown in panels **a** and **b**. The labels "b" and "d" designate octahedra from "bright" row and "dim" row, respectively.

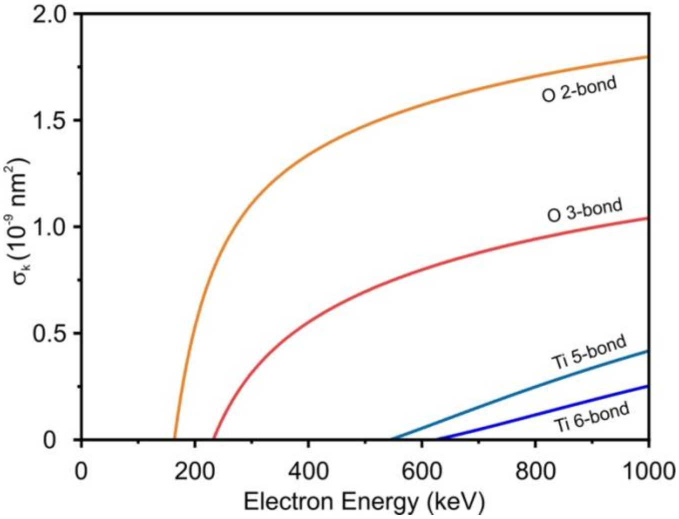


**Supplementary Figure 14. Surface sputtering probabilities for O and Ti atoms in rutile $TiO_2$.** The knock-on cross-sections were calculated using the Mott cross-section for relativistic incident electron: $\sigma_k(E_0) = 4\pi a_0^2 \left(\frac{Z^2 U_R{}^2}{(m_0c^2)^2}\right) \frac{1-\beta^2}{\beta^4} \times [(\xi - 1) - \beta^2\, ln(\xi) + \pi\alpha\beta \left\{2\left[\xi^{\frac{1}{2}} - 1\right] - \ln(\xi - 1)\right\}]$, where $a_0$ is the Bohr radius, $Z$ is the number of moving unit, $U_R$ is Rydberg energy, $m_0$ is the rest mass of electron, $c$ is the speed of light, and $\beta = \frac{\mathrm{v}}{\mathrm{c}} = \sqrt{1 - \left(1 + \frac{E_0}{m_0c^2}\right)^{-2}}$, $\alpha = \frac{Z}{137}$, $\xi = E_{max}/E_{th}^{(2)}$, $E_{max} = \frac{2E_0(E_0+2m_0c^2)}{M_0c^2}$, $M_0$ is the mass of the atom, $E_{th}^{(2)}$ is the threshold energy that must be transferred to sputter atoms from the surface.[9,10] Ti 6-bond atomic displacement energy and O 3-bond atomic displacement energy are 47 eV and 39 eV, respectively.[11,12]

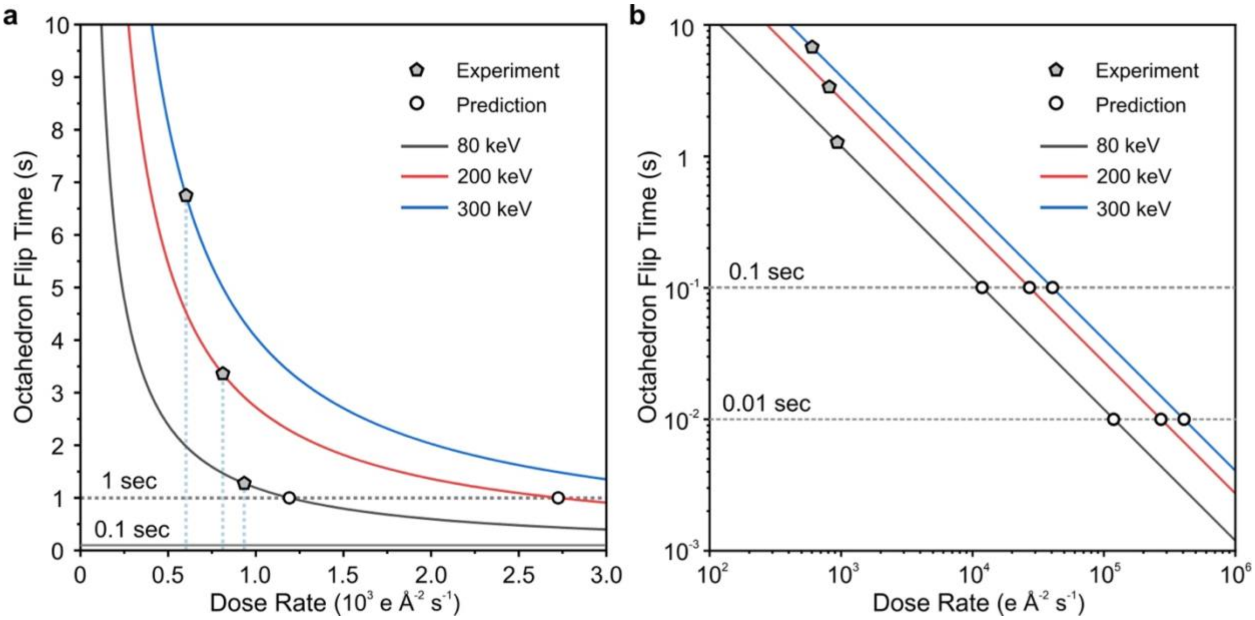


**Supplementary Figure 15. Octahedron flip time vs dose rate for electron energies: 80, 200, 300 keV. a,** Experimental data points are following dose rates: 934, 812, and 602 e $Å^{-2}$ $s^{-1}$ for 80, 200 and 300 keV, respectively, shown with the grey data indicators. The 1-second dashed line shows intersections with the 80 and 200 keV curves at dose rates of 1194 and 2734 e $Å^{-2}$ $s^{-1}$. The 0.1-second horizontal line indicates that the dose rate needs to be very high to achieve "octahedra" flipping. **b,** The octahedron flip time vs dose rate plotted in a wider range. The 0.1 second dashed line shows intersections with the 80, 200, and 300 keV lines at dose rates of $1.1\times10^4$, $2.5\times10^4$ and $3.8\times10^4$ e $Å^{-2}$ $s^{-1}$. The 0.01 second dashed line shows intersections with the 80, 200, and 300 keV lines at dose rates of $1.2\times10^5$, $2.5\times10^5$ and $3.7\times10^5$ e $Å^{-2}$ $s^{-1}$.

**Supplementary Table 1. Calculation of the cohesive energies for different wide bandgap metal oxides.** In these calculations the values of heat of formation, $\Delta H_f^0$, and heat of atomization ($\Delta H_M^0$ for metal and $\Delta H_O^0$ for oxygen) were used to evaluate the cohesive energies, $E_{coh}$, for the $MO_2$ unit and for single M-O bond [13-19].

| Materials ($MO_2$) | Heat of formation ($\Delta H_f^0$) (eV/$MO_2$) | Heat of atomization ($\Delta H_M^0$) (eV/atom) | Heat of atomization ($\Delta H_O^0$) (eV/atom) | $E_{coh}$ (eV/$MO_2$) | $E_{coh}$ (eV/bond) |
|---|---|---|---|---|---|
| Rutile-$TiO_2$ | 9.79 [13] | 4.88 [14] | 2.6 [14] | 19.9 | 3.3 |
| Anatase-$TiO_2$ | 9.72 [13] | 4.88 [14] | 2.6 [14] | 19.8 | 3.3 |
| Rutile-$GeO_2$ | 5.59 [15] | 3.907 [14] | 2.6 [14] | 14.7 | 2.5 |
| Rutile-$SnO_2$ | 5.98 [16] | 3.14 [14] | 2.6 [14] | 14.3 | 2.4 |
| Rutile-$IrO_2$ | 2.58 [17] | 6.954 [14] | 2.6 [14] | 14.7 | 2.5 |
| $\alpha$-quartz $GeO_2$ | 5.59 [15] | 3.907 [14] | 2.6 [14] | 14.7 | 3.7 |
| $\alpha$-quartz $SiO_2$ | 9.44 [18] | 4.73 [14] | 2.6 [14] | 19.7 | 4.9 |
| $\alpha$-$Al_2O_3$ (sapphire) | 17.37 ($Al_2O_3$) [19] | 3.39 [14] | 2.6 [14] | 31.9 ($Al_2O_3$) | 2.7 |

**Supplementary Table 2. Summary of the exciton energies and their lifetimes for different wide bandgap metal oxides.** The values of bandgap and cohesive energies, determined theoretically and experimentally (Supplementary Table 1), are also listed for evaluation of viability of radiolysis in these materials.[20-37]

| Materials | Building unit | Exciton energy (eV) | Exciton lifetime ($\tau_{ex}$) | Band gap (eV) | $E_{coh}$ (eV/bond) | |
|---|---|---|---|---|---|---|
| | | | | | Theoretical | Experimental |
| Rutile-$TiO_2$ | Octahedral | 2.91, 3.05 [20,21] | 16 ns [22] | 3.0 [22] | 3.57 [23] | 3.3 |
| Anatase-$TiO_2$ | Octahedral | 2.91, 3.26 [21] | 0.1-1 ns [22] | 3.2 [22] | 3.59 [24] | 3.3 |
| Rutile-$GeO_2$ | Octahedral | 4.64, 4.67 [25] | -- | 4.68 [25] | 2.41 [26] | 2.5 |
| Rutile-$SnO_2$ | Octahedral | 3.2, 3.6 [27,28] | 0.6 ns [28] | 3.64 [27] | 2.58 [29] | 2.4 |
| Rutile-$IrO_2$ | Octahedral | -- | -- | Metallic | 2.8 [29] | 2.5 |
| α-quartz $GeO_2$ | Tetrahedral | 6.6 [30] | -- | 5.5-6 [30] | 3.45 [26] | 3.7 |
| α-quartz $SiO_2$ | Tetrahedral | 8-11 [31] | 14 ns [32] | 7.52-9.6 [31] | 5-5.5 [33] | 4.9 |
| α-$Al_2O_3$ (sapphire) | Octahedral | 7.6 [34]<br>9.1[35] | 20-150 ns [34]<br>-- | 8.80 [36] | 2.59 [37] | 2.7 |

## Supplementary references


1 Kirkland, E. J. *Advanced Computing in Electron Microscopy*. (Springer, New York, 2010), 2nd Edition

2 Sears, V. F. & Shelley, S. A. Debye-Waller Factor for Elemental Crystals. *Acta Crystallographica Section A* **47**, 441-446 (1991).

3 Bertolotti, F., Vivani, A., Moscheni, D., Ferri, F., Cervellino, A., Masciocchi, N. & Guagliardi, A. Structure, Morphology, and Faceting of $TiO_2$ Photocatalysts by the Debye Scattering Equation Method. The P25 and P90 Cases of Study. *Nanomaterials* **10** 743 (2020).

4 Jeong, J. S., Odlyzko, M. L., Xu, P., Jalan, B. & Mkhoyan, K. A. Probing core-electron orbitals by scanning transmission electron microscopy and measuring the delocalization of core-level excitations. *Phys. Rev. B* **93** 165140 (2016).

5 Egerton, R. F. *Electron Energy-Loss Spectroscopy in the Electron Microscope*. Second edn, (Plenum Press, New York and London), 2nd Edition.

6 Basha, A., Levi, G., Amrani, T., Li, Y., Ankonina, G., Shekhter, P., Kornblum, L., Goldfarb, I. & Kohn, A. Elastic and inelastic mean free paths for scattering of fast electrons in thin-film oxides. *Ultramicroscopy* **240** 113570 (2022).

7 Raether, H. *Excitations of Plasmons and Interband Transitions by Electrons*. (Springer-Verlag Berlin Heidelberg GmbH, 1980), vol. 88.

8 Hobbs, L. W. Introduction to analytical electron microscopy, edited by J. J. Hren, J. I. Goldstein, and D. C. Joy. (Scanning Microscopy International, Chicago, 1979), p. 437.

9 Mott, N. F. The scattering of fast electrons by atomic nuclei. *Proceedings of the Royal Society of London Series a-Containing Papers of a Mathematical and Physical Character* **124**, 425-442 (1929).

10 Mckinley, W. A. & Feshbach, H. The Coulomb Scattering of Relativistic Electrons by Nuclei. *Phys. Rev.* **74**, 1759-1763 (1948).

11 Buck, E. C. Effects of Electron-Irradiation of Rutile. *Radiat Eff. Defects Solids* **133**, 141-152 (1995).

12 Smith, K. L., Colella, M., Cooper, R. & Vance, E. R. Measured displacement energies of oxygen ions in titanates and zirconates. *J. Nucl. Mater.* **321**, 19-28 (2003).

13 Chase, M. W., Curnutt, J. L., Prophet, H., Mcdonald, R. A. & Syverud, A. N. JANAF thermochemical tables, 1975 supplement. *J. Phys. Chem. Ref. Data* **4**, 1 (1975).

14 Kittel, C. *Introduction to Solid State Physics*. (John Wiley & Sons, New York, NY, 2004) 8th edn.

15 Jolly, W. L. & Latimer, W. M. The Equilibrium Ge(S) + $GeO_2$(S) = 2GeO(G) - the Heat of Formation of Germanic Oxide. *J. Am. Chem. Soc.* **74**, 5757-5758 (1952).

16 Wagman, D. D., Wagman, D. P., Cox, J. D. & Medvedev, V. A. *CODATA Key Values for Thermodynamics*. *CODATA Key Values for Thermodynamics* (Hemisphere, New York, 1984) 1st Edition.

17 Cordfunke, E. H. P. The Enthalpy of Formation of $IrO_2$ and Thermodynamic Functions. *Thermochim. Acta* **50**, 177-185 (1981).

18 Chase, M. W. *NIST-JANAF Themochemical Tables, Fourth Edition*. *NIST-JANAF Thermochemical Tables*, (American Institute of Physics, 1998) Monograph 9, 4th Edition..

19 Chase, M. W., Curnutt, J. L., Mcdonald, R. A. & Syverud, A. N. Janaf Thermochemical Tables, 1978 Supplement. *J. Phys. Chem. Ref. Data* **7**, 793-940 (1978).

20 Amtout, A. & Leonelli, R. Time-Resolved Photoluminescence from Excitons in $TiO_2$. *Solid State Commun.* **84**, 349-352 (1992).
21 Kernazhitsky, L., Shymanovska, V., Gavrilko, T., Naumov, V., Fedorenko, L., Kshnyakin, V. & Baran, J. Room temperature photoluminescence of anatase and rutile $TiO_2$ powders. *J. Lumin.* **146**, 199-204 (2014).
22 Yamada, Y. & Kanemitsu, Y. Determination of electron and hole lifetimes of rutile and anatase $TiO_2$ single crystals. *Appl. Phys. Lett.* **101** 133907 (2012).
23 Glassford, K. M. & Chelikowsky, J. R. Structural and Electronic-Properties of Titanium-Dioxide. *Phys. Rev. B* **46**, 1284-1298 (1992).
24 Lazzeri, M., Vittadini, A. & Selloni, A. Structure and energetics of stoichiometric $TiO_2$ anatase surfaces (vol 63, art no 155409, 2001). *Phys. Rev. B* **63** 155409 (2002).
25 Stapelbroek, M. & Evans, B. D. Exciton Structure in UV-Absorption Edge of Tetragonal $GeO_2$. *Solid State Commun.* **25**, 959-962 (1978).
26 Chae, S., Lee, J., Mengle, K. A., Heron, J. T. & Kioupakis, E. Rutile $GeO_2$: An ultrawide-band-gap semiconductor with ambipolar doping. *Appl. Phys. Lett.* **114** 102104 (2019).
27 Reimann, K. & Steube, M. Experimental determination of the electronic structure of $SnO_2$. *Solid State Commmun.* **105**, 649-652 (1998)..
28 Liu, R. B., Chen, Y. J., Wang, F. F., Cao, L., Pan, A. L., Yang, G. Z., Wang, T. H. & Zou, B. S. Stimulated emission from trapped excitons in $SnO_2$ nanowires. *Physica E Low Dimens. Syst. Nanostruct.* **39**, 223-229 (2007)
29 Hamad, B. A. First-principle calculations of structural and electronic properties of rutile-phase dioxides ($MO_2$), M = Ti, V, Ru, Ir and Sn. *EPJ B* **70**, 163-169 (2009)..
30 Trukhin, A. N. Luminescence of a Self-Trapped Exciton in $GeO_2$ Crystal. *Solid State Commun.* **85**, 723-728 (1993).
31 Trukhin, A. N. Excitons in $SiO_2$ - a Review. *J Non-Cryst Solids* **149**, 32-45 (1992).
32 Hughes, R. C. Charge-Carrier Transport Phenomena in Amorphous $SiO_2$ - Direct Measurement of Drift Mobility and Lifetime. *Phys. Rev. Lett.* **30**, 1333-1336 (1973).
33 Teter, D. M., Gibbs, G. V., Boisen, M. B., Allan, D. C. & Teter, M. P. First-Principles Study of Several Hypothetical Silica Framework Structures. *Phys. Rev. B* **52**, 8064-8073 (1995).
34 Kirm, M., Zimmerer, G., Feldbach, E., Lushchik, A., Lushchik, C. & Savikhin, F. Self-trapping and multiplication of electronic excitations in $Al_2O_3$ and $Al_2O_3$ : Sc crystals. *Phys. Rev. B* **60**, 502-510 (1999)
35 Loh, E. Ultraviolet reflectance of $A1_2O_3$, $SiO_2$ and BeO. *Solid State Commun.* **2**, 269-272 (1964).
36 Santos, R. C. R., Longhinotti, E., Freire, V. N., Reimberg, R. B. & Caetano, E. W. S. Elucidating the high-k insulator alpha-$Al_2O_3$ direct/indirect energy band gap type through density functional theory computations. *Chem. Phys. Lett.* **637**, 172-176 (2015).
37 Janetzko, F., Evarestov, R. A., Bredow, T. & Jug, K. First-principles periodic and semiempirical cyclic cluster calculations for single oxygen vacancies in crystalline $Al_2O_3$. *Phys. Status Solidi B* **241**, 1032-1040 (2004).
