# Supplementary material for: Mending cracks atom-by-atom in rutile TiO2 with electron beam radiolysis
Source: Nat Commun. 2023 Sep 26;14:6005. doi: 10.1038/s41467-023-41781-x (PMC10522652; doi:10.1038/s41467-023-41781-x)
Supplement: Supplementary file 3 — Description of Additional Supplementary Files [file 41467_2023_41781_MOESM3_ESM.pdf]

Supplementary Movie 1. Crack's restructuring with accumulation of electron doses. Each high-resolution HAADF-STEM image frame was acquired under dose rate of 812 e $Å^{-2}$ s^{-1} and the scan time for each frame is 80.5 s. With the accumulation of electron doses, these frames show the gradually filling of the crack with rutile TiO_2 crystal. The full frame width is 39 nm. Frames are low-pass filtered for noise reduction.

Supplementary Movie 2. Illustration of "2-step rolling" mechanism. First panel shows octahedral units in "bright" row rolling from left to right, while the second panel shows octahedral unit in "dim" row rolling from left to right. The third panel shows octahedral unit rolling vertically from up to down along the edge.

Supplementary Movie 3. Crack restructuring with accumulation of electron doses under different electron energies. Three movies are taken under 80, 200, and 300 keV beam electron energies. HAADF-STEM image frames were acquired at dose rate of 934, 812, and 602 e $Å^{-2}$ s^{-1} for 80, 200, and 300 keV, respectively. The scan time for each frame was 80.5 s. The full frame width in each case is 31 nm. Frames are low-pass filtered for noise reduction.
